# Editorial and Miscellaneous

**Published:** 1865-10

**Authors:** 


### EDITORIAL AND MISCELLANEOUS.

### RUSH MEDICAL COLLEGE.

The Twenty-Third Annual Course of Lectures was opened on the 4th instant. The general Introductory Address was delivered by Prof. Blaney, who had been absent on duty in the army during the past three years. The Lecturer, after welcoming the large class of students present on the occasion, referred in feeling and appropriate terms to the happy conclusion of hostilities within our borders, and paid a merited tribute to the valor of the soldiers of the Union, and the efficiency of the Medical staff of the army. He then admonished those just commencing the study of Medicine, of the great labor necessary to acquire a knowledge of the principles of the profession—instancing the importance and extent of research necessary to master the individual branches, as Anatomy, Physiology, Pathology, Surgery, Practical Medicine, Therapeutics, &c., portions of which were strikingly beautiful and pertinent. The address, as a whole, was well conceived, and abounded in good advice to the young men of the class, and was admirably calculated to raise the respect for our noble profession in the minds of all who were fortunate enough to hear it.

The course opens with every prospect of being among the most successful and satisfactory which have latterly attended the history of the College.

*Messrs. Editors Chicago Medical Journal*: Surgeon Winch, in concluding his excellent paper on Chronic Diarrhœa in the August number of the JOURNAL, speaks of the use of Nux

Vomica in that disease. My preceptor, Dr. J. T. Boyd, of Indianapolis, used it very successfully in his practice. And I have myself, in a number of cases of returned soldiers, prescribed it with very great benefit.

The cases in which it seemed to be most especially indicated and in which I have seen most benefit follow its use, were those characterized by anæmia, loss of appetite, indigestion, and great debility. In some cases no other remedies were used—excepting, of course, the proper dietetic measures—and with well-marked success. Most of these cases had been previously treated in the army, from three to six months, and even longer. And as the continuation of astringents, opiates, and in some cases, purgatives, had utterly failed, I concluded it would be useless to persue that course longer. After taking the Nux Vomica for a short time—generally from two to three weeks—the appetite returns, digestion is more perfectly carried on, the patient gains strength, and the discharges gradually become more consistent and less frequent. The *rationale* of its cure I will not pretend to give—but merely to state that the experience I have had with it in a considerable number of cases, is very much in its favor, and would urge for it a trial. I have, in all cases, used the tincture. Will not some one give to the profession the benefit of a more extended experience?

David R. Johnston, M. D.

Muncie, Ind., Sept. 7th, 1865.

---

We have received from the publishers the following works. Want of space prevents a notice in this number:

The Student's Book of Cutaneous Medicine and Diseases of the Skin. By Erasmus Wilson, F. R. S.

Lectures on Diseases of the Stomach. By William Brinton, M. D., F. R. S. Lea & Blanchard.

The Use of the Laryngoscope in Diseases of the Throat. By Morill Mackenzie, M. D. Lindsay & Blakiston.

Physician's Visiting List for 1866. Lindsay & Blakiston.

